# Nomogram model of survival prediction for nasopharyngeal carcinoma with lung metastasis: developed from the SEER database and validated externally

**DOI:** 10.3389/fonc.2024.1351578

**Published:** 2024-03-19

**Authors:** Zhehao Xiao, Kaiguo Li, Fang Su, Xiaohui Yang, Hongxing Zou, Song Qu

**Affiliations:** ^1^ Department of Radiation Oncology, Affiliated Tumor Hospital of Guangxi Medical University, Nanning, China; ^2^ Key Laboratory of Early Prevention and Treatment of Regional High Incidence Tumors, Ministry of Education, Guangxi Medical University, Nanning, China; ^3^ Nasopharyngeal Carcinoma Clinical Medical Research Center, Guangxi Medical University, Nanning, China

**Keywords:** nasopharyngeal carcinoma, lung metastasis, nomogram, prognosis, SEER database

## Abstract

**Objective:**

Distant metastasis occurs in some patients at the first diagnosis of nasopharyngeal carcinoma (NPC), the prognosis is poor, and there are significant individual differences. This study established a nomogram model of lung metastasis of NPC as a supplement to TNM staging.

**Methods:**

The training cohort is used to build the nomogram model, and the validation cohort is used to evaluate the model. The training cohort of 177 patients is from the Surveillance, Epidemiology, and End Results (SEER) database. Factors affecting overall survival (OS) in patients with lung metastasis of NPC analysis by Cox regression analysis and then a nomogram were established. 122 patients from the Affiliated Tumor Hospital of Guangxi Medical University were selected as the external validation cohort. The concordance index (C-index), the area under the curve (AUC), and the calibration curve were used to assess the accuracy of the nomogram and used the decision curve analysis (DCA) curve to measure the clinical benefit capacity of the model. The patients were separated into two groups with different risks, and the “Kaplan-Meier (KM)” survival analysis was used to evaluate the differentiation ability of the model.

**Results:**

Age, T-stage, radiation, chemotherapy, and brain metastases can affect the OS in NPC with lung metastasis. A nomogram was developed according to the above five factors. The C-index of the training cohort and the validation cohort were 0.726 (95% CI: 0.692-0.760) and 0.762 (95% CI: 0.733-0.791). The AUC of the nomogram was better than that of the TNM staging. In the training cohort, the nomogram predicted OS AUC values of 0.767, 0.746, and 0.750 at 1, 2, and 3 years, TNM stage of 0.574, 0.596, and 0.640. In the validation cohort, nomogram predictions of OS AUC values of 0.817, 0.857, and 0.791 for 1, 2, and 3 years, TNM stage of 0.575, 0.612, and 0.663. DCA curves suggest that nomogram have better clinical net benefits than TNM staging. The KM survival analysis shows that the nomogram has a reasonable risk stratification ability.

**Conclusion:**

This study successfully established a nomogram model of NPC lung metastasis, which can be used as a supplement to TNM staging and provide reference for clinicians.

## 1 Introduction

Nasopharyngeal carcinoma (NPC) is an epithelial carcinoma originating from the inner lining of the nasopharyngeal mucosa. It is highly aggressive and most commonly occurs in the pharyngeal recess of the nasopharynx (1). Based on the International Agency for Research on Cancer (IARC), there were about 133 thousand new cases of NPC, representing 0.7 percent of the amount of all cancer cases diagnosed in 2020 (2). NPC has unique geographical distribution characteristics; 70% of cases are diagnosed in East and Southeast Asia, especially in southern China. It is recognized that the epidemic of NPC is connected with the infection of Epstein-Barr virus (EBV) (3–5), and other risk factors include dietary habits, environmental influences (6), and genetic susceptibility (7). Recently, Luo proposed the refreshingly NPC ecology theory, which posits cancer as a multidimensional spatiotemporal “unity of ecology and evolution “pathological ecosystem. The cancer ecological theory can help us to open a new perspective and better understand the complex process of NPC development and metastasis (8). In the era of intensity-modulated radiotherapy (IMRT), the therapeutic effect of NPC patients with low clinical stage has been significantly improved (9–11). However, due to the early clinical symptoms being vague and difficult to distinguish and the invasion and metastasis of tumor cells being strong, some patients are already in the advanced stage when they are diagnosed. About 4-15% of patients already had metastases when they were first diagnosed with NPC (12), and the survival of these patients is often highly variable and unsatisfactory. Currently, TNM staging is used to predict the survival of NPC. However, patients with distant metastases were classified as stage M1 (13).The same stage may have different survival outcomes. TNM staging has limited predictive value, and more effective and accurate predictive tools may be needed.

NPC is prone to lung metastasis (14). Some studies have shown that NPC with metastasis limited to the lung may have better overall survival (OS) than those with other sites of metastasis (15, 16). However, this conclusion has not been confirmed by large-scale studies, and the majority of patients with lung metastasis also have metastasis to other sites. In 2011, Cao et al. (17) developed risk subsets containing some clinical variables to predict the survival rate of lung metastasis of NPC patients. However, the results need to be visualized more, and clinical detection methods were limited at that time. Some patients with small metastases of lung may be missed, which may bias the study results. Therefore, the survival prognosis of these patients needs further study.

Surveillance, Epidemiology, and End Results (SEER) is a public database and research resource created by the National Cancer Institute (18). This database collects and stores data on cancer incidence, survival, and therapies across America to support research and epidemiological investigations. In this study, a prognostic model of NPC with lung metastasis was established through the data of the SEER database, and the model was externally verified by patients from the Affiliated Tumor Hospital of Guangxi Medical University to help doctors predict the survival of these patients individually and provide guidance for decision-making.

## 2 Materials and methods

### 2.1 Patient selection

The training cohort is used to build the nomogram model, and the validation cohort is used to evaluate the practicality and reliability of the model. Training cohort data for this study was obtained from SEER*Stat software, version 8.4.2, and it is rooted in the Incidence-SEER Research Data of 17 Registries, Nov 2022 Sub (2000–2020). Inclusion criteria: (1) Year of diagnosis from 2010 to 2017. (2) site recode referring to the International Classification of Diseases-Oncology, Third Edition (ICD-O-3) or World Health Organization (WHO) 2008=“Nasopharynx”. (3) Behavior code ICD-O-3 = “Malignant”. (4) Diagnostic confirmation = “Microscopically confirmed”. (5) SEER Combined Mets at DX-lung = “Yes”. Exclusion criteria: (1) Age below 18 years at diagnosis. (2) The diagnosis source is “Autopsy only” or “Death certificate only”. (3) Clinical data were missing, or follow-up information was incomplete. Validation cohort data came from patients of the Affiliated Tumor Hospital of Guangxi Medical University from 2015 to 2021, and the patient screening criteria were identical to the training cohort.

### 2.2 Data collection

Baseline information was collected for all patients, including age at diagnosis, sex, marital status, race, pathological type, primary site, T-stage status, N-stage status, surgery, chemotherapy, radiation, liver metastasis, bone metastasis, brain metastasis, survival status, and OS. Pathological diagnosis codes included the most common type, nonkeratinizing squamous cell carcinoma (NKC) (8072, 8073) and others (8020, 8021, 8082, 8083, 8070, 8071,8074, 8075, 8010). All patients were staged with the 7th edition of the American Joint Committee on Cancer (AJCC) staging system, and the deadline for follow-up in the validation cohort was November 15, 2023. This study has passed the review of the Ethics Committee (Review number: KY2024021).

### 2.3 Statistical analysis

Continuous variables used the Mann-Whitney U test, recorded as median with interquartile range. Categorical variables used the Chi-square test, recorded as numbers with proportions (%). Univariate Cox regression was used to forecast the impact of a single factor on OS, and variables with statistical significance were included in multivariate Cox regression analysis to identify independent impact factors of OS. P < 0.05 was considered statistically significant. Subsequently, a nomogram was established for patients with NPC with lung metastasis. This nomogram can predict a patient’s 1, 2, and 3-year OS by calculating the total points for each patient.

Concordance index (C-index) and area under curve (AUC) were used to judge the predictive ability of the nomogram and compared with the traditional TNM staging. Calibration curves are used to assess the correctness of the nomogram. DCA curve to assess the clinical net benefit capacity of the nomogram model and contrast it with the TNM stage system. Finally, we obtained the total points of each patient and used X-tile software to obtain the best cut-off points to separated into two groups with different risks. “Kaplan-Meier(KM)” curve is drawn for survival analysis. All statistical analyses were performed using SPSS (version 25.0) and R (version 4.3.1) software.

## 3 Results

### 3.1 Patient characteristics

**Figure 1:**
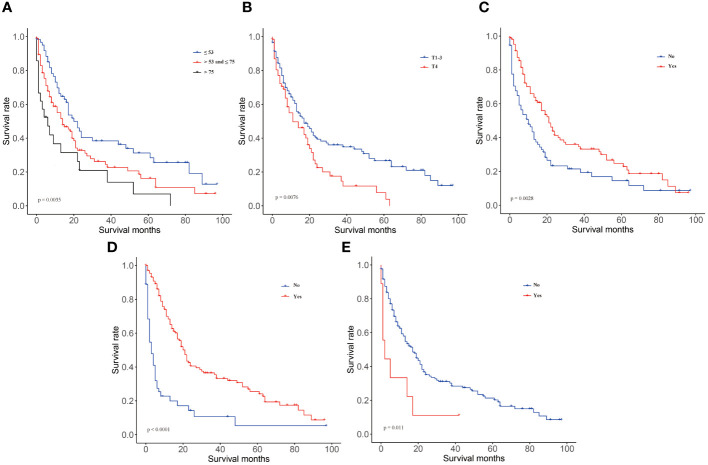
KM curves in training group of OS. **(A)** age, **(B)** T-stage, **(C)** radiotherapy, **(D)** chemotherapy, **(E)** brain metastases.

We included 177 patients in the training cohort and 122 validation cohort. All of these patients were diagnosed with lung metastasis of NPC. 131 patients died in the training cohort and 84 in the validation cohort. There were no statistically significant differences between the two cohorts on factors other than age and race (**Table 1**). In univariate Cox regression analysis, we found that age, radiation, T-stage, chemotherapy, and brain metastases could be correlated with patients’ OS. Therefore, we included the above factors in the multivariate analysis. Subsequent result indicated that these factors were independent key factors affecting patients’ OS (**Table 2**). Older, higher T-stage and brain metastases were linked to worse OS in patients, and accepting chemotherapy and radiotherapy were linked to better OS in patients (**Figure 1**).

**Table 1:** Basic characteristics of patients.

Factors		Training cohort	Validation cohort	p value
Age		60 (49-70)	47 (40-55)	<0.001
Sex	Male	132 (74.6)	96 (78.7)	0.411
	Female	45 (25.4)	26 (21.3)	
Marital status	Married	93 (52.5)	75 (61.5)	0.126
	Others	84 (47.5)	47 (38.5)	
Race	Black	28 (15.8)	0 (0)	<0.001
	Others	85 (48.0)	122 (100)	
	White	64 (36.2)	0 (0)	
T-stage	T1	33 (18.6)	12 (9.8)	0.153
	T2	24 (13.6)	14 (11.5)	
	T3	59 (33.3)	45 (36.9)	
	T4	61 (34.5)	51 (41.8)	
N-stage	N0	22 (12.4)	16 (13.1)	0.051
	N1	54 (30.5)	27 (22.1)	
	N2	63 (35.6)	36 (29.5)	
	N3	38 (21.5)	43 (35.3)	
Histologic type	NKC	152(85.9)	110 (90.2)	0.286
	Others	25 (14.1)	12 (9.8)	
Primary site	NOS	143 (80.8)	98 (80.3)	0.921
	Others	34 (19.2)	24 (19.7)	
Radiation	No	72 (40.7)	46 (37.7)	0.605
	Yes	105 (59.3)	76 (62.3)	
Surgery	No	167 (94.4)	121 (99.2)	0.062
	Yes	10 (5.6)	1 (0.8)	
Chemotherapy	No	45 (25.4)	37 (30.3)	0.350
	Yes	132 (74.6)	85 (69.7)	
Bone mets	No	113 (63.8)	65 (53.3)	0.067
	Yes	64 (36.2)	57 (46.7)	
Brain mets	No	168 (94.9)	118 (96.7)	0.452
	Yes	9 (5.1)	4 (3.3)	
Liver mets	No	137 (77.4)	85 (69.7)	0.133
	Yes	40 (22.6)	37 (30.3)	
Survival status	Alive	46 (26.0)	38 (31.1)	0.329
	Dead	131 (74.0)	84 (68.9)	

**Table 2:** Univariate and multivariate cox regression analysis of OS in patients with lung metastasis of NPC.

Factors		Univariate analysis	p value	Multivariate analysis	p value
		HR (95%CI)		HR (95%CI)	
Age		1.020 (1.007-1.027)	0.001	1.023 (1.012-1.033)	<0.001
Sex	female	Reference			
	male	1.383 (0.978-1.957)	0.067		
Marital status	Married	Reference			
	Others	1.107 (0.821-1.491)	0.506		
Race	Black	Reference			
	Others	0.780 (0.508-1.198)	0.256		
	White	0.923 (0.590-1.444)	0.725		
T-stage	T1	Reference			
	T2	1.453 (0.854-2.474)	0.168	1.442 (0.835-2.489)	0.189
	T3	1.303 (0.845-2.008)	0.231	1.525 (0.975-2.383)	0.064
	T4	1.946 (1.257-3.013)	0.003	2.420 (1.518-3.857)	<0.001
N-stage	N0	Reference			
	N1	1.231 (0.744-2.036)	0.419		
	N2	1.097 (0.672-1.790)	0.711		
	N3	0.925 (0.545-1.570)	0.773		
Histologic type	NKC	Reference			
	Others	0.670 (0.438-1.024)	0.064		
Primary site	NOS	Reference			
	Others	1.246 (0.855-1.816)	0.252		
Radiation	No	Reference			
	Yes	0.717(0.530-0.970)	0.031	0.656(0.476-0.905)	0.010
Surgery	No	Reference			
	Yes	0.719(0.379-1.365)	0.313		
Chemotherapy	No	Reference			
	Yes	0.391(0.276-0.555)	0.001	0.332(0.230-0.480)	<0.001
Bone mets	No	Reference			
	Yes	1.206(0.881-1.650)	0.243		
Brain mets	No	Reference			
	Yes	2.271(1.155-4.465)	0.017	1.780(1.092-3.631)	0.010
Liver mets	No	Reference			
	Yes	1.361(0.954-1.942)	0.090		

### 3.2 Nomogram establishment

**Figure 2:**
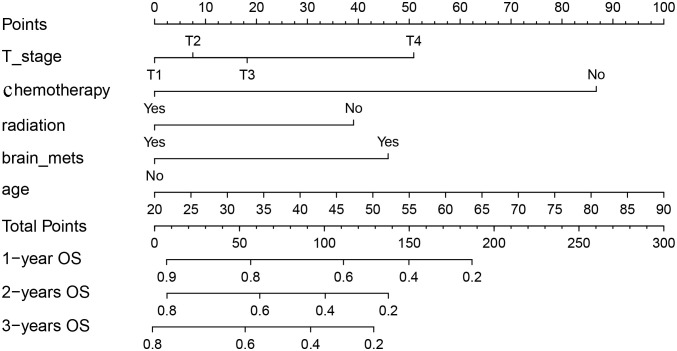
Nomogram for survival prediction of patients with lung metastasis of NPC.

We included above five factors in the nomogram for predicting OS: age, T-stage, radiation, chemotherapy, and brain metastases. Each patient is given a point for each factor, corresponding to the “Points”axis, to get a point based on that factor. Finally, the respective points based on the five factors are added together to get a total points, corresponding to the “Total points” axis, the 1, 2, and 3-year OS probability of the patient can be predicted (**Figure 2**). For example, a 50-year-old (42.7 points), T3(18.3 points) patient with lung metastasis of NPC, received chemotherapy (0 points) and radiotherapy (0 points) and did not have brain metastases (0 points) had a total point of 61.0. The patient’s 1, 2 and 3-year survival rate was 75%, 62%, and 54%.

### 3.3 Nomogram evaluation and verification

**Figure 3:**
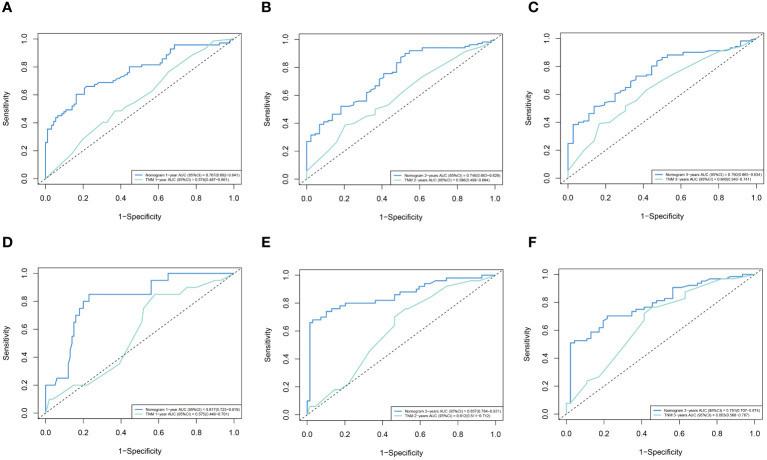
ROC curves of the nomogram and TNM stage. **(A)** training cohort 1 year. **(B)** training cohort 2 years. **(C)** training cohort 3 years. **(D)** validation cohort 1 year. **(E)** validation cohort 2 years. **(F)** validation cohort 3 years.

We analyze the C-index and AUC of the model to assess the model’s predictive ability. It is generally believed that the C-index value is equal to 0.5, indicating that the model has no predictive ability. If it is between 0.5 and 0.7, the power is low. Moreover, if it is between 0.7 and 0.9, the model has moderate accuracy. A C-index value larger than 0.9 indicates that the model has high prediction accuracy. Using the Bootstrap self-sampling method, set Bootstrap =1000 times, the model was verified, and the C-index in the training cohort was 0.726 (95% CI: 0.692-0.760) and 0.762 (95% CI: 0.733-0.791) in the validation cohort, suggesting that the model has moderate prediction accuracy. Similarly, the AUC also suggests this nomogram has great power. In the two cohorts, the AUC of the nomogram was better than that of the TNM stage. The nomogram predicted OS AUC values of 0.767, 0.746, and 0.750 at 1, 2, and 3 years, which were significantly higher than those of 0.574, 0.596, and 0.640 in the TNM stage in the training cohort. We found remarkable consistency in the validation cohort, with nomogram predictions of OS AUC values of 0.817, 0.857, and 0.791 for 1,2, and 3 years, better than those for TNM stages of 0.575, 0.612, and 0.663 (**Figure 3**).

**Figure 4:**
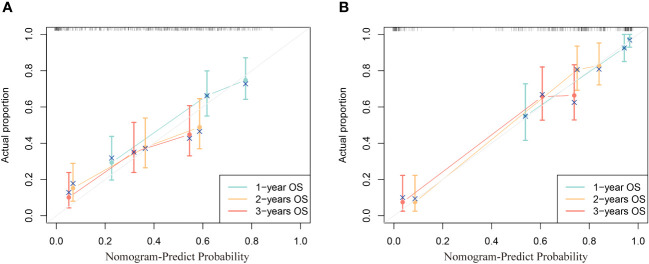
Calibration curves of the nomogram. **(A)** training cohort and **(B)** validation cohort.

The calibration curve reflects the agreement from the probability of the actual to the probability of the predicted. In the calibration curve, the closer to the diagonal line, the higher its consistency. It can be seen from the calibration curve that the prediction 1, 2, and 3-year OS calibration curves in two cohorts all have a high coincidence with the diagonal line, indicating that this nomogram is accurate (**Figure 4**).

**Figure 5:**
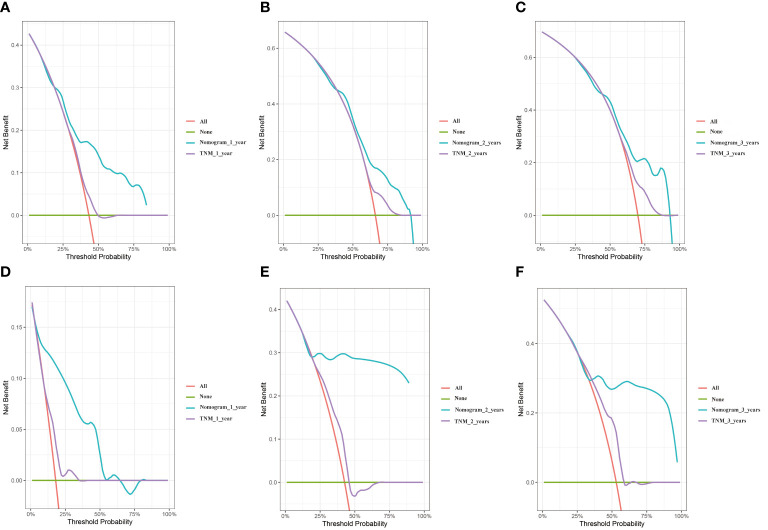
DCA curves of the nomogram and TNM stage. **(A)** training cohort 1 year. **(B)** training cohort 2 years. **(C)** training cohort 3 years. **(D)** validation cohort 1 year. **(E)** validation cohort 2 years. **(F)** validation cohort 3 years.

Decision curve analysis (DCA) is an approach to assess the degree of patient benefit; by introducing “threshold probability,” medical intervention is triggered under the same threshold probability. If the net benefit brought by the column graph to the patient is high, its clinical significance is high. Practicality will be better. The DCA curves of this prediction model are located in the upper right portion of both extreme cases, where the green line is all negative (net benefit is zero) and the red line is all positive (net benefit is negative of the slope). The net benefit of the nomogram was evaluated by drawing DCA curves to predict 1, 2, and 3-year OS in two cohorts and contrast to the TNM stage system. We clearly found that the nomogram prediction model we created was compared to the TNM stage system, which has better clinical benefits and shows reasonable clinical practicability in two cohorts (**Figure 5**).

**Figure 6:**
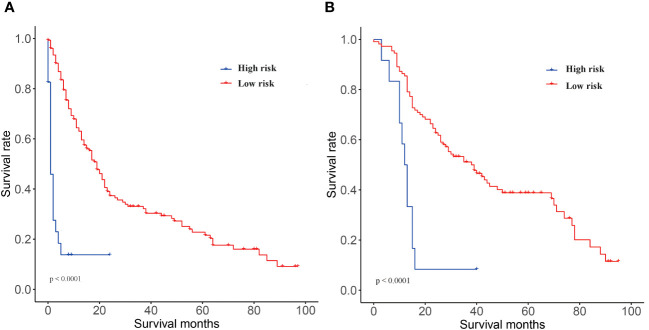
KM curves of OS based on the nomogram risk stratification. **(A)** training cohort and **(B)** validation cohort.

To estimate the predictive power of the nomogram for stratification of patient risk, we summarized the total points for each patient, with the training group (17.14-283.98) and the validation cohort (15.32-252.41). X-Tile software was used to obtain the optimal cut-off point of 197.6 for the total points of the training cohort, and the patients were divided in two groups: “low-risk” (total points ≤ 197.6) and “high-risk” (total points > 197.6) for KM survival analysis. The results suggested that patients in the “low-risk” group had a better OS compared to patients in the high-risk group (p < 0.0001) (**Figure 6**). The results further verify that the nomogram can stratify the risk of NPC with lung metastasis, which is beneficial for further guidance of follow-up treatment.

## 4 Discussion

The treatment of distant metastasis patients is essential to improve the efficacy of NPC, which substantially affects the NPC patients ‘survival rate (19). The TNM staging system can only forecast the survival of some patients. It has been shown that patients in the same TNM stage could have distinct outcomes after getting a similar treatment, while patients with metastasis are mainly at the same stage. Therefore, developing an individualized and accurate model for prognosis judgment and therapy is highly desirable. Studies have shown that patients with different metastatic sites and states may have different prognoses. The median OS in patients with single-organ metastatic NPC was better than in patients with extensive metastasis (24.8 months vs 12.8 months, P < 0.001) (20). Huang et al. showed that the prognosis of NPC patients with brain metastases was poor, and the 3-year OS rate was 16.3% (21). Qu et al. proposed that liver metastases were associated with poor cancer-specific survival (CSS) in patients (22). Hui et al. found that nasopharyngeal carcinoma patients with distant metastatic limited to the lung had good OS compared with patients with metastasis from other sites (16). Therefore, we focused on lung metastasis of NPC. This study is the first nomogram for NPC with lung metastasis. Nowadays, nomograms are widely used. Using Cox regression analysis, Wu et al. developed a nomogram for predicting OS in patients with low-grade endometrial stromal sarcoma (23). Zheng et al. developed a nomogram for predicting OS in lung cancer bone metastases patients (24). Nomogram can visualize the results of our study and has good practicability (25, 26).This study indicated that being older was related to worse OS in patients, the same as the conclusions of many researchers. Lin et al. (27) suggested age was linked to poor OS in locoregionally advanced NPC patients. Li et al. (28) also pointed out that NPC patients’ CSS gradually decreases with age, and there is a gender difference, which peaks at 55-60 years of age. This conclusion has also been confirmed in other cancer, and Zheng et al. (24) confirmed that increasing age is related to worse OS in lung cancer bone metastasis. Cancer is considered an age-related disease, and it is generally thought that people over the age of 65 are more likely to have cancer (29). At the same time, due to aging, physiological reserves are reduced, which may lead some patients unable to tolerate anti-tumor therapy. For example, reduced clearance of some cytotoxic chemotherapeutic drugs that need to be excreted by the kidneys will make it difficult for elderly patients to undergo anti-tumor therapy, which will also shorten their OS (30). Mashiro et al. found that chemotherapy-induced neutropenia (CIN) has been related to age > 65 (31). Therefore, additional attention may need to be paid to older NPC patients, and further research in the future may focus on specific treatment options for such patients to improve their prognosis.

Brain metastasis of NPC is not common. It is still in the sporadic reporting stage, and the related metastasis pathway has not been fully clarified. Tumor cells enter the Cerebro-Spinal Fluid (CSF) by corroding the skull and destroying the dural membrane, which may be one of the ways of brain metastasis of NPC (32). Brain metastases in tumors are generally regarded as a marker of poor prognosis (33), and in our nomogram, it is also associated with poor OS in NPC patients. Unfortunately, there is currently no consensus on diagnosing brain metastasis in NPC. In some cases, individual patients have been reported to have achieved a good survival prognosis through chemoradiotherapy (34) (35). Although brain metastases from NPC are rare, they cannot be ignored, and these patients may need more mature treatment options to help them survive for a long time.

We found that NPC patients with lung metastases who received chemotherapy and radiotherapy had better survival than those who did not. Currently, according to the results of several multi-center clinical trials, including the CAPTAIN 1ST, JUPITER-02, and RATIONALE 309, PD-1 inhibitor combined with chemotherapy has become the first-line standard treatment for recurrent/metastatic nasopharyngeal cancer (R/M NPC) (36–38). However, these patients have lost the opportunity for radical radiotherapy because of distant metastases. You et al. initiated a phase 3 Randomized Clinical Trial, which indicated that the 2-year OS was longer in the chemotherapy combined with radiotherapy group than in the chemotherapy alone group (54.5% vs. 76.4%) (HR=0.42; 95%CI: 0.23-0.77; p = 0.004) (39). This finding has also been seen in retrospective studies. Liu et al. (40) found that 1-year progression-free survival (80.6% vs. 65.1%, P<0.001) and OS (98.3% vs. 89.5%, P=0.001) were significantly better in patients with immunochemotherapy and radiotherapy than with metastatic who received immunotherapy and chemotherapy. The coalescence of chemotherapy and radiotherapy has a significant synergistic effect. In the cell cycle, the action point of radiotherapy to kill cancer cells is the late G2, M, and G1, and does not affect the S stage. In contrast, the main action point of some chemotherapy drugs, such as gemcitabine, is the S stage so that radiotherapy can play a complementary killing role on chemotherapy-resistant cancer cells (41, 42). In addition, radiation therapy can also produce a “distant effect”, that is the phenomenon of regression of metastatic tumors far from the irradiation field after local irradiation (43). The distant effect may be related to immune activation, such as increased PD⁃L1 expression, lymphocytopenia, and induced aggregation of immunosuppressive cells (44). Unfortunately, further analysis was not possible due to the lack of information on patient immunotherapy.

Overall, our model is successful, as it is composed of 5 common factors, including T-stage, radiation, chemotherapy, age, and brain metastases, and achieves good survival prediction in NPC patients with lung metastases compared to traditional TNM staging. Although the training cohort was based on patients from America, our results show that the model is also suitable for patients in China. However, there is no denying that this study has some shortcomings. At first, more detailed baseline data of patients, such as EBV DNA copy number, chemoradiotherapy regimen, immunotherapy, comorbidities, and Karnofsky performance status (KPS) score of patient status, could not be obtained, so further detailed analysis could not be carried out. Second, the age in the two cohorts has a statistical difference, in which patients in the training cohort were more concentrated in age. However, this may not have obviously biased the results. Finally, with a fraction of patients in the validation cohort diagnosed in 2021 and a follow-up cutoff of less than three years, predictions of three-year long-term survival have yet to have much chance to prove their value.

## 5 Conclusion

In this study, a survival and prognosis model for patients with NPC with lung metastasis was established, which can be used as a supplement to TNM staging, help clinicians analyze the prognosis of these patients individually, and support a simple tool for treatment decision-making.

## Data availability statement

The datasets presented in this study can be found in online repositories. The names of the repository/repositories and accession number(s) can be found in the article/supplementary material.

## Ethics statement

The studies involving humans were approved by Affiliated Tumor Hospital of Guangxi Medical University. The studies were conducted in accordance with the local legislation and institutional requirements. The ethics committee/institutional review board waived the requirement of written informed consent for participation from the participants or the participants’ legal guardians/next of kin because This is a retrospective study. Some of the data comes from public databases, and these data are public resources. The other part of the data is the collection or study of previously archived data, documents, materials, and information that is recorded in a way that the investigator cannot contact the patient.

## Author contributions

ZX: Data curation, Formal analysis, Methodology, Software, Writing – original draft. KL: Data curation, Formal analysis, Writing – original draft. FS: Data curation, Formal analysis, Writing – original draft. XY: Data curation, Formal analysis, Writing – original draft. HZ: Data curation, Formal analysis, Writing – original draft. SQ: Writing – review & editing.
